# The effect of metformin on the survival of colorectal cancer patients with type 2 diabetes mellitus

**DOI:** 10.1038/s41598-022-16677-3

**Published:** 2022-07-20

**Authors:** Zeinab Tarhini, Kamelia Manceur, Julien Magne, Muriel Mathonnet, Jeremy Jost, Niki Christou

**Affiliations:** 1 Laboratory INSERM U1308, CAPTuR, Control of Cell Activation in Tumor Progression and Therapeutic Resistance, Medical School, 2 rue du Docteur Marcland, 87025 Limoges Cedex, France; 2 Inserm U1094, IRD U270, Univ. Limoges, CHU Limoges, EpiMaCT - Epidemiology of chronic diseases in tropical zone, Institute of Epidemiology and Tropical Neurology, OmegaHealth, Limoges, France; 3 Centre d’Epidémiologie, de Biostatistique et de Méthodologie de la Recherche-CEBIMER, CHU de Limoges-BMA, 2 rue Martin Luther King, 87042 Limoges, France; 4 Digestive Surgery Department, University Hospital of Limoges, Avenue Martin Luther King, 87000 Limoges, France; 5 Clinical Pharmacy Unit, Pharmacy Department, University Hospital of Limoges, Avenue Martin Luther King, 87000 Limoges, France

**Keywords:** Cancer, Drug discovery, Endocrinology, Gastroenterology, Oncology

## Abstract

Evidence from previous studies suggests a protective effect of metformin in patients with colorectal cancer (CRC). The aim of this study was to examine the associations between metformin use and overall survival (OS) and disease-free survival (DFS) in CRC patients with type 2 diabetes mellitus (DM). We retrospectively included patients who underwent surgery for CRC at Limoges’ University Hospital between 2005 and 2019 and diagnosed with type 2 DM. Data on the characteristics of patients, CRC, comorbidities and drug exposure were collected from the electronic medical records. The exposure was the use of metformin and the outcomes were OS and DFS. We identified 290 CRC patients with type 2 DM. A total of 144 (49.7%) of them were treated with metformin. Metformin users were significantly younger, with higher body mass index and less diabetes-related complications compared to non-users. The 2-year OS was significantly higher in metformin users than in non-users (86.9 ± 2.9% vs. 71.0 ± 4.0%, p = 0.001). In multivariate analysis, metformin use was associated with better OS (adjusted hazard ratios [aHR] = 0.45 95% confidence interval [95% CI]: 0.21–0.96) and better DFS (aHR = 0.31; 95% CI: 0.18–0.54). In conclusion, the use of metformin may improve OS and DFS in CRC patients with type 2 DM.

## Introduction

Colorectal cancer (CRC) is the second most deadly cancer worldwide (9.4%), and the third most diagnosed form of cancer globally (10.0%) for both sexes combined^1^. In 2020, nearly 2 million people were diagnosed with CRC, with about 935,000 CRC-related deaths^2^. The reference treatment of CRC is surgery. However, even after surgical removal, the recurrence rate of CRC remains high^3^.

The prevalence of Diabetes Mellitus (DM) in CRC patients varied between 2.8 and 14% in studies from various countries^4^. However, among Taiwanese patients, two studies showed higher prevalence (17% and 23.6%)^4,5^. Metformin, a biguanide class agent, is an antihyperglycemic drug for type 2 DM. It lowers blood glucose concentrations without causing hypoglycemia by inhibiting hepatic gluconeogenesis and by reducing peripheral insulin resistance^6^.

In addition to its antidiabetic effect, it has been shown that metformin has an antineoplastic effect and can inhibit cancer cell growth^7^. The protective effect of metformin against cancer has been reported in previous studies such as breast cancer^8^, lung cancer^9^, ovarian cancer^10^, renal cell carcinoma^11^.

The results of the association between metformin use and the risk of recurrence in CRC are not entirely consistent^12^. A large registry-based study was conducted in diabetic CRC patients to evaluate the effect of metformin on CRC survival. They found no association between metformin use and disease-free survival (DFS) or recurrence-free survival after adjustment for multiple confounders. However, only patients treated medically for diabetes were included and classified as diabetic^12^.

Another meta-analysis was performed to evaluate whether metformin could improve survival in CRC patients with type 2 diabetes. They found that metformin use was associated with increased overall survival (OS) rate and CRC-specific survival^13^. However, the study had several limitations, including the heterogeneity between the studies, in term of differences in CRC stage and length of follow-up, and no assessment of the impact of diabetes severity in patients on insulin^13^.

Given the inconsistency of the literature's results on the effect of metformin on CRC survival, which may be related to the heterogeneity of the population in terms of demographics and clinical presentation, we intended to evaluate the effect of metformin in our population.

Our objective was to examine the association between metformin use in CRC patients with type 2 DM and OS and DFS at two years after surgery for CRC.

## Materials and methods

### Study design

The present study was retrospective cohort including patients who underwent surgery for CRC in the department of digestive, general and endocrine surgery at Limoges University Hospital-France between January 01, 2005 and April 30, 2019 and diagnosed with type 2 DM.

### Study population

#### Inclusion and exclusion criteria

Patients older than 18 years were eligible if they had undergone surgery for CRC and diagnosed with type 2 DM. Patients who were excluded were those with type 1 diabetes, benign colorectal tumor, non-CRC after anatomopathological examination (appendix cancer, colonic and rectal metastases from another primary cancer), non-resection of the colorectal tumor during surgery, patients under trusteeship and patients with incomplete records on anatomic pathology examination and medications.

### Diabetes assessment and antidiabetic drug exposure

In our study, type 2 DM was defined as medically- or diet-treated diabetes. Patients in the "diet-treated diabetes" group were defined as patients with type 2 DM who did not receive a prescription for an antidiabetic medication (oral or injectable drugs) for their type 2 DM.

Patients in the "medically-treated diabetes" group were defined as receiving at least one antidiabetic drug within the Anatomical Therapeutic Chemical (ATC) groups: A10BA02 (metformin), A10A (insulin and its analogues), A10BB (sulfonylureas), A10BF (alpha-glucosidase inhibitor inhibitors), A10BJ (Glucagon Like Peptide-1 analogues), A10BH (Dipeptidyl peptidase-4 inhibitors) or A10BX (repaglinide).

#### Exposure definition

Diabetic patients were classified into two groups: metformin users and non-users. In our study, we defined the exposure to metformin as taking oral metformin for at least 90 days during the follow-up period after surgery^14,15^. The prescription for metformin as well as the duration of use were found in the drug prescription software of patients included in the hospital.

For the metformin group, metformin could be used alone or in combination with other antidiabetic drugs. In the metformin non-users group, patients could be treated either by diet alone or by any other antidiabetic drug than metformin.

### End point: survival outcomes

Survival outcomes were compared between metformin users and non-users in patients with type 2 DM. The primary end point was overall survival (OS), defined as the time from surgery for CRC to death from any cause. Mortality was determined by the date of death using the death certificate through the deceased patient's medical record.

Disease-free survival (DFS) was analyzed as a secondary outcome and defined as the time from CRC surgery to the first documented local or distant recurrence (metastasis) of CRC, development of a new primary colorectal tumor or death from any cause within the two years after CRC surgery.

Local recurrence was defined as recurrence in the anastomosis site or in the pelvic cavity structure (vagina, bladder, and lymph nodes located in the pelvic cavity). Distant recurrence was defined as recurrence in the systemic lymph nodes, liver, lungs, peritoneum, bones, and brain^16^. The diagnosis of local or distant tumor recurrence of CRC was determined by computed tomography (CT) scan or magnetic resonance imaging (MRI) and verified by biopsy.

Histopathology of the biopsy was used to identify the origin of the cancer. Suspected cases were analyzed by expert (NC) in order to confirm the presence or not of recurrence of CRC. Regarding patients followed outside our hospital after CRC surgery, the presence of recurrent CRC was assessed using biological and imaging results sent to their specialists at the Limoges University Hospital.

### Follow-up time

The dates of patient entry into the cohort were the dates for which each patient underwent CRC surgery; the dates of exit were the earliest date among the dates of death, recurrence of CRC or development of a new primary colorectal tumor within the two years after surgery, the date of last consultation (in case of relocation or lost to follow-up) or the date of end of the follow-up (two years after the study enrollment).

### Data collection

The data collection took place over four months. It was started on January 1, 2021 and completed on April 30, 2021. The electronic medical records (EMR) of each patient were used to collect the data through the hospital computerized patient record softwares. The data were coded anonymously and entered manually into a secured database.

### Variables

The following variables were collected:

- Sociodemographic parameters: age at surgery, sex (male, female), body mass index (BMI), family history of cancer and CRC.
- Cancer treatment: surgery alone, surgery and adjuvant chemotherapy, neoadjuvant treatment and surgery.
- Clinical parameters for CRC:
- Tumor site classified into colon cancer and rectal cancer. Rectal cancer includes the rectum. Colon cancer includes: the right colon (consists of caecum, ascending colon, and right hepatic flexure), transverse colon (encompasses the segment of colon between right hepatic flexure and left hepatic flexure as it is described by anatomists), left colon (consists of descending colon, left hepatic flexure and sigmoid colon) and rectosigmoid junction).
- CRC TNM stage at diagnosis (in situ, I, II, III, IV) according to AJCC 8th edition^17^, type of CRC (Lieberkühn adenocarcinoma, mucinous adenocarcinoma, medullary carcinoma, neuroendocrine carcinoma), tumor histology (well differentiated, moderately differentiated, poorly differentiated, undifferentiated), tumor size (< 4, ≥ 4 cm), microsatellite status [microsatellite instability (MSI) or microsatellite stable tumor (MSS)], type of mutation (*KRAS, BRAF, NRAF*, no mutations).
- Medication use: Metformin use (alone/in combination), use of other antidiabetic medications, diet-treated diabetes, comedication (antihypertensive drugs, hypolipidemic drugs), number of antidiabetics and drugs/day.
- Diabetes: diabetes with complications (yes/no), and glycemic control (controlled diabetes if glycosylated hemoglobin (HbA1c) ≤ 7% and uncontrolled if HbA1c > 7%).
- Charlson comorbidity index (CCI score): this score was used to assess the patients' comorbidities. It contains 19 criteria in which each disease is assigned a score according to its influence on the mortality. We classified the score into three levels (0, 1–2, > 2)^12,18^.
- Tumor markers: included carbohydrate antigen levels (CA19-9 positive > 39 U/mL), carcinoembryonic antigen levels (CEA positive ≥ 5 ng/mL).

### Statistical analysis

The normality of quantitative variables was tested using the Kolmogorov–Smirnov test.

Data were presented as mean ± standard deviation (SD) for continuous variables and median (interquartile, IQR) for skewed variables. Qualitative variables were summarized using frequency and percentages. Comparative analysis was carried out using Pearson's Chi-square test or Fisher's exact test for categorical variables and Student *t* test or Mann–Whitney for quantitative variables. Survival curves were obtained using the Kaplan–Meier method, and the survival curves of each group were compared by a log-rank test. Multivariate Cox proportional Hazard models were performed to estimate the adjusted hazard ratios (HR) and their corresponding 95% confidence intervals (95% CI) for the association between metformin use and the outcomes (OS, DFS).

A p-value < 0.05 was considered statistically significant and all p-values were 2-sided. All analyses were performed using Statistical Package for the Social Sciences (SPSS) version 22.0 software.

#### Covariates: assessment of potential confounders

The selection of the potential confounders in the multivariate analysis was based on clinical expertise and literature review. The covariates used were age at surgery, sex, BMI, CRC stage, Charlson comorbidity index, tumor site and diabetes complications.

### Ethics statement

The approval for this study was obtained by the Ethics Committee of the University Hospital of Limoges (No. 479-2021-135). Informed consent was obtained from all patients. All methods were performed in accordance with the relevant guidelines and regulations (Strobe guidelines for observational studies).

## Results

**Figure 1:**
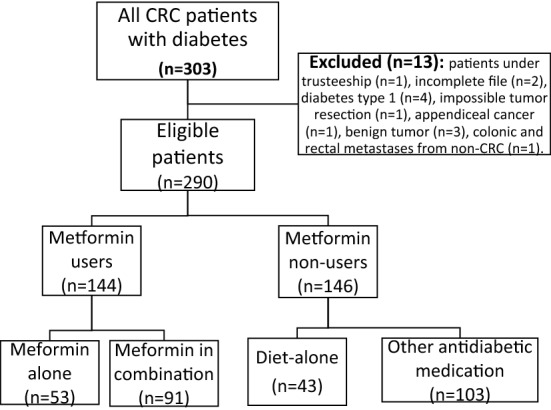
Flowchart showing the process of enrollment of colorectal cancer patients.

A flowchart outlining the process of enrollment of CRC patients is presented below in Fig. 1. After exclusion (n = 13), 290 patients operated for CRC between 2005 and 2019 at Limoges university hospital were identified with type 2 DM (Fig. 1).

### Patients’ characteristics

Among the 290 CRC patients with type 2 DM, 144 (49.7%) were treated with metformin: either metformin alone (36.8%) or metformin in association with other antidiabetic drugs (63.2%). Among metformin non-users (n = 146, 50.3%), 43 (29.4%) were treated by diet alone (Fig. 1).

The main demographic and clinical characteristics and medication use of patients are summarized in Tables 1 and 2. The mean age of patients who had surgery for CRC was 73.71 ± 8.70 years, 69.3% were males and 32.4% were overweight. Of all the patients in this study who have CRC, 75.2% had colon cancer and the rest had rectal cancer (24.8%). In addition, 55.8% of patients were diagnosed at stages Tis, I and II and 43.5% were diagnosed at stages III and IV (Table 2).

**Table 1:** Main characteristics of CRC patients with type 2 diabetes mellitus compared by metformin status. Significant values are in bold.

Variables	All patients (n = 290)	Metformin non-users (n = 146)	Metformin users (n = 144)	p-value
**Age**, years	73.71 ± 8.70	75.05 ± 8.72	72.35 ± 8.53	**0.008**
**Male sex**, n (%)	201 (69.3)	95 (65.1)	106 (73.6)	0.12
BMI*, kg/m^2^	27.2 [25.0–31.9]	26.8 [24.0–30.9]	27.7 [25.6–32.7]	**0.02**
Charlson comorbidity index*	1.0 [0.0–2.0]	1.0 [0.0–3.0]	1.0 [0.0–2.0]	0.10
**Complications of diabetes**, n (%)	37 (12.8)	25 (17.1)	12 (8.3)	**0.03**
**Glycemic control, n (%)**				0.23
Yes (HbA1c ≤ 7%)	136 (46.9)	66 (56.9)	70 (64.8)	
Missing	66 (22.8)			
Number of drugs/day*	7.0 [5.0–9.0]	7.0 [5.0–9.3]	7.0 [5.0–9.0]	0.82
Number of antidiabetics/day*	1.0 [1.0–2.0]	1.0 [0.0–1.0]	2.0 [1.0–2.0]	**< 0.001**
**Metformin use**, n (%)	144 (49.7)			
**Medications, n (%)**				
Insulin use	78 (26.9)	50 (34.2)	28 (19.4)	**0.004**
Sulfonylureas use	87 (30.0)	43 (29.5)	44 (30.6)	0.84
GLP-1 analogues use	7 (2.4)	2 (1.4)	5 (3.5)	0.28
AGIs inhibitors use	16 (5.5)	5 (3.4)	11 (7.6)	0.12
DDP-4 inhibitors use	44 (15.2)	14 (9.6)	30 (20.8)	**0.008**
Other glucose-lowering drugs use	18 (6.2)	11 (7.5)	7 (4.9)	0.35
Diet-alone	43 (14.8)			
Antihypertensive drugs	234 (80.7)	117 (80.1)	117 (81.3)	0.81
Hypolipidemic drugs	147 (50.7)	65 (44.5)	82 (56.9)	**0.03**
Chemotherapy	103 (35.5)	48 (32.9)	55 (38.2)	0.34
Neoadjuvant treatment	37 (12.8)	16 (11.0)	21 (14.6)	0.36

*Indicates variables that are not normally distributed.

Quantitative variables are reported as mean ± SD or median [IQR].

**Table 2:** Main characteristics of CRC patients with type 2 diabetes mellitus compared by metformin status (continued).

Variables	All patients (n = 290)	Metformin non-users (n = 146)	Metformin users (n = 144)	p-value
**Tumor site, n (%)**				0.69
Right colon	120 (41.4)	63 (43.2)	57 (39.6)	
Colon transverse	12 (4.1)	7 (4.8)	5 (3.5)	
Left colon	62 (21.4)	32 (21.9)	30 (20.8)	
Rectum	72 (24.8)	35 (24.0)	37 (25.7)	
Rectosigmoid junction	24 (8.3)	9 (6.2)	15 (10.4)	
**CRC stage, n (%)**				0.19
In situ	11 (3.8)	8 (5.5)	3 (2.1)	
I	52 (17.9)	24 (16.6)	28 (19.6)	
II	99 (34.1)	47 (32.4)	52 (36.4)	
III	95 (32.8)	54 (37.2)	41 (28.7)	
IV	31 (10.7)	12 (8.3)	19 (13.3)	
Missing	2 (0.7)			
**Type of cancer, n (%)**				0.99
Lieberkühn adenocarcinoma	271 (93.4)	136 (93.2)	135 (93.8)	
Mucinous adenocarcinoma	15 (5.2)	8 (5.6)	7 (4.9)	
Medullary carcinoma	3 (1.0)	1 (0.7)	2 (1.4)	
Neuroendocrine carcinoma	1 (0.3)	1 (0.7)	0 (0.0)	
Family history of cancer, n (%)	45 (15.5)	22 (15.1)	23 (16.0)	0.83
Family history of CRC, n (%)	25 (8.6)	15 (10.3)	10 (6.9)	0.31
**Tumor size, cm**				0.40
≥ 4	160 (55.2)	76 (55.9)	84 (60.9)	
Missing	16 (5.5)			
**Histological type, n (%)**				0.71
Well differentiated	49 (16.9)	28 (23.0)	21 (16.9)	
Moderately differentiated	181 (62.4)	87 (71.3)	94 (75.8)	
Poorly differentiated	14 (4.8)	6 (4.9)	8 (6.5)	
Undifferentiated	2 (0.7)	1 (0.8)	1 (0.8)	
Missing	44 (15.2)			
**Type of mutation, n (%)**				0.84
KRAS	26 (9.0)	12 (34.3)	14 (35.9)	
BRAF	9 (3.1)	4 (11.4)	5 (12.8)	
NRAS	4 (1.4)	1 (2.9)	3 (7.7)	
No mutations	35 (12.1)	18 (51.4)	17 (43.6)	
Missing	216 (74.5)			
**Microsatellite status, n (%)**				0.84
MSI	12 (4.1)	6 (21.4)	6 (19.4)	
MSS	47 (16.2)	22 (78.6)	25 (80.6)	
Missing	231 (79.7)			
**CEA positive**, ≥ 5 ng/mL, n (%)	76 (26.2)	41 (39.4)	35 (30.7)	0.18
**CA 19-9 positive**, > 39 U/mL, n (%)	28 (9.7)	11 (12.5)	17 (16.2)	0.47

### Comparison between metformin users and non-users

Metformin non-users were significantly older than users (p = 0.008), had lower BMI (p = 0.02) and more diabetes complications (p = 0.03). In addition, there was a significant higher proportion of insulin use within the metformin non-users group (p = 0.004, Table 1).

### Outcomes

**Figure 2:**
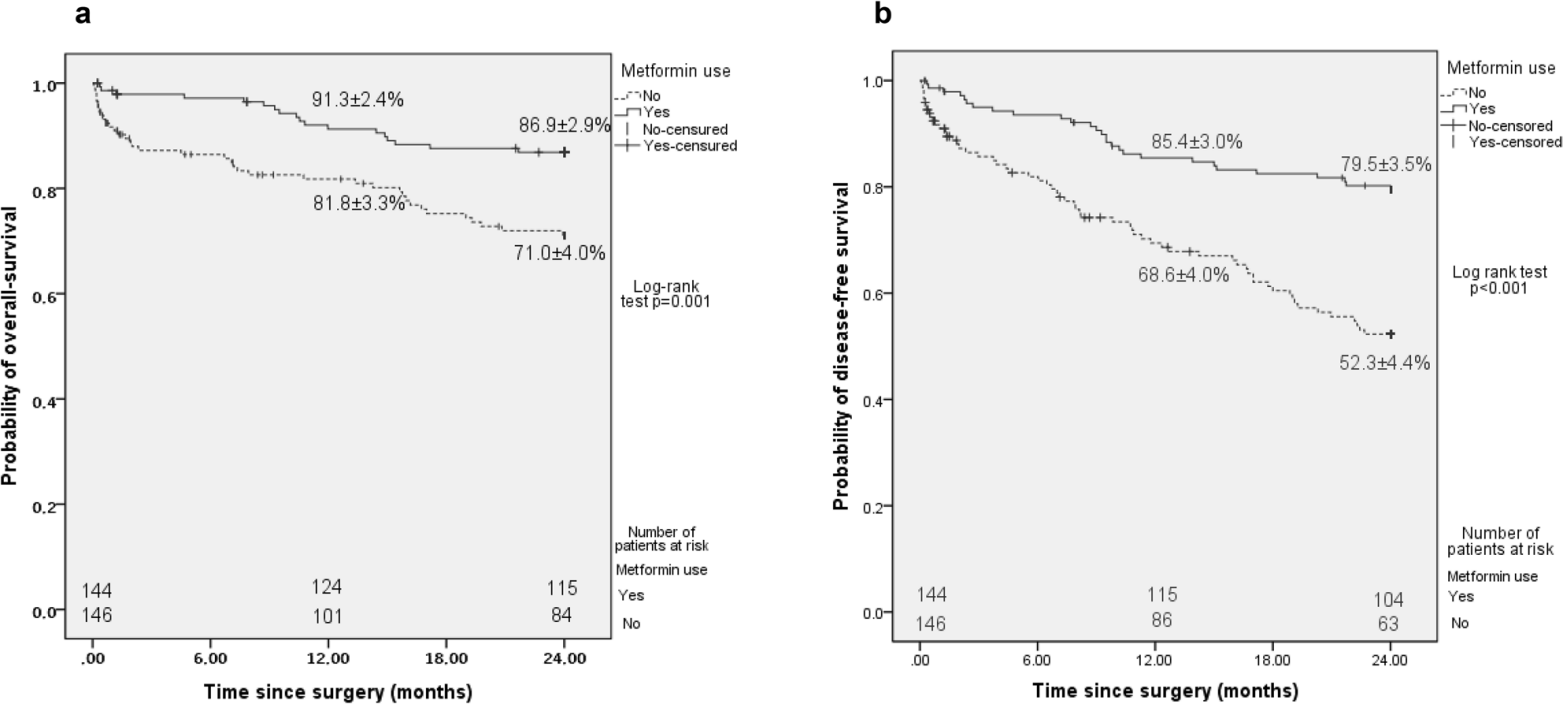
Survival curves comparing metformin users and non-users. (**a**) Overall survival, (**b**) disease-free survival.

During a mean follow-up of 18.94 ± 8.62 months, 56 (19.3%) death and 54 (18.6%) recurrence of CRC, resulting in a 2-year OS and DFS of 79.2 ± 2.5% and 66.3.8 ± 2.9% respectively. As compared to metformin non-users (including patients with diet-treated diabetes), patients receiving metformin depicted significant higher 2-year OS (71.0 ± 4.0% vs. 86.9 ± 2.9%, p = 0.001, Fig. 2a). In addition, metformin users had significantly improved 2-year DFS than metformin non-users (79.5 ± 3.5% vs. 52.3 ± 4.4%, p < 0.001, Fig. 2b).

### Multivariate analyses

On multivariate analysis, after adjustment for confounding factors, Cox modeling showed that metformin was associated with better OS (adjusted Hazard Ratio [aHR] = 0.45; 95% Confidence Interval [95% CI]: 0.21–0.96; p = 0.04, Table 3). Others independent determinants of OS were age (aHR = 1.10; 95% CI: 1.05–1.16), Stages III, IV CRC (aHR = 4.64; 95% CI: 2.11–10.22, Table 3).

**Table 3:** Overall survival assessed by the Cox regression model. Significant values are in bold.

Variables	Overall-survival					
	HR	95% CI	p-value	aHR^a^	95% CI	p-value
**Age at surgery**, years	1.11	1.07–1.15	< 0.001	1.10	1.05–1.16	**0.001**
**Female sex**	1.14	0.65–1.99	0.66			
**BMI,** kg/m^2^	0.95	0.89–1.02	0.14			
**Tumor site**						
Colon (reference)						
Rectum	0.52	0.25–1.06	0.07			
**CRC stage**						
In situ, I, II (reference)						
III, IV	3.64	2.04–6.51	< 0.001	4.64	2.11–10.22	**0.001**
**Charlson comorbidity index**	1.21	1.04–1.40	**0.01**			
**Complications of diabetes**	2.45	1.32–4.55	**0.005**			
**Metformin use**	0.40	0.23–0.71	**0.001**	0.45	0.21–0.96	**0.04**

HR, hazard ratio; CI, confidence interval.

^a^Adjusted for age at surgery, sex, BMI, tumor site, CRC stage, Charlson comorbidity index, diabetes complication.

In addition, metformin use was associated with better DFS (aHR = 0.31; 95% CI: 0.18–0.54, Table 4). The independent determinants of DFS were age at surgery (aHR = 1.03; 95% CI: 1.00–1.07), female sex (aHR = 0.48; 95% CI: 0.23–0.98), and stages III, IV CRC (aHR = 3.15, 95% CI: 1.85–5.36).

**Table 4:** Disease-free survival assessed by the Cox regression model. Significant values are in bold.

Variables	Disease-free survival					
	HR	95% CI	p-value	aHR^a^	95% CI	p-value
**Age at surgery**, years	1.05	1.02–1.07	**0.001**	1.03	1.00–1.07	**0.04**
**Female sex**	0.87	0.54–1.38	0.55	0.48	0.23–0.98	**0.04**
**BMI**, kg/m^2^	0.96	0.91–1.00	0.06			
**Tumor site**						
Colon (reference)						
Rectum	0.61	0.36–1.04	0.07			
**CRC stage**						
In situ, I, II (reference)						
III, IV	3.23	2.08–5.01	**< 0.001**	3.15	1.85–5.36	**< 0.001**
**Charlson comorbidity index**	1.21	1.08–1.36	**0.001**			
**Complications of diabetes**	2.31	1.39–3.84	**0.001**			
**Metformin use**	0.36	0.23–0.56	**< 0.001**	0.31	0.18–0.54	**< 0.001**

HR, hazard ratio; CI, confidence interval.

^a^Adjusted for age at surgery, sex, BMI, tumor site, CRC stage, Charlson comorbidity index, diabetes complications.

### Subgroup analyses

**Figure 3:**
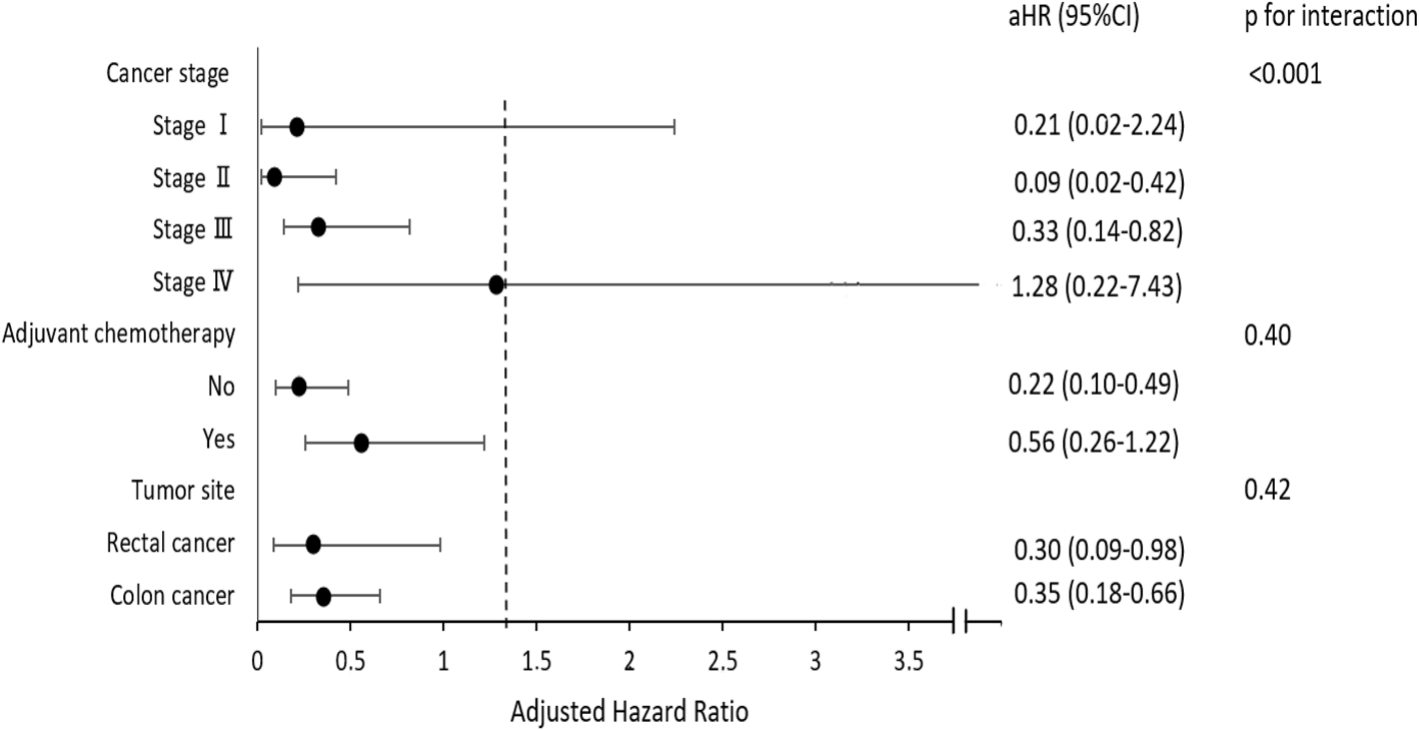
Forest plot for subgroup analysis: effect of metformin on disease-free survival.

Figure 3 summarized the effect of metformin on DFS in different subgroups in multivariate analysis. The use of metformin was associated with better DFS in stage II CRC (aHR = 0.09; 95% CI: 0.02–0.42), stage III (aHR = 0.33; 95% CI: 0.14–0.82), colon cancer (aHR = 0.35; 95% CI: 0.18–0.66) and rectal cancer subgroup (aHR = 0.30; 95% CI: 0.09–0.98, Fig. 3). Furthermore, among metformin users, having a BMI ≥ 25 had no effect on DFS compared with a normal BMI (aHR = 0.43; 95% CI: 0.17–1.09). In multivariate analysis, the use of metformin in combination with other antidiabetic drugs was not associated with OS (aHR = 2.17, 95% CI: 0.49–9.59) nor DFS (aHR = 0.68, 95% CI: 0.23–2.01) compared with metformin users alone.

### Comparison of the survival of metformin users according to their microsatellite status

Among patients with MSS, metformin users had an improved DFS compared to non-users (62.3 ± 10.0% vs. 22.2 ± 9.8%; p = 0.03). However, there was no significant differences between metformin users and non-users in patients with MSI (83.3 ± 15.2% vs. 80.0 ± 17.9%; p = 0.95).

Among metformin users with colon cancer and MSS, there was no significant difference in DFS between patients who received adjuvant chemotherapy and those who did not (50.0 ± 15.8% vs. 72.9 ± 16.5%; p = 0.31). However, among metformin users with MSI and colon cancer, there was a significant difference in DFS between patients who received adjuvant chemotherapy and those who did not (p = 0.05). In addition, among patients with rectal cancer and MSS who used metformin, there was no significant difference in DFS between those who received neoadjuvant treatment and those who did not (p = 0.09).

### Stratified analysis

In stratified analysis by age of 74 years, metformin users have a better DFS compared to non-users in both age categories; younger and older than 74 years separately (aHR = 0.40; 95% CI: 0.18–0.88 and aHR = 0.30; 95% CI: 0.13–0.69 respectively).

## Discussion

This study evaluated the association between metformin use and survival of CRC patients with type 2 DM. Our results showed that metformin-users have better OS and DFS with a 55% decrease in all-cause mortality at 2-year after surgery for CRC. Our findings were consistent with previous results conducted by Ramjeesingh et al*.* in 2016 at the Southeastern Ontario Cancer Center (2-year OS: 80.5% for the metformin group vs. 67.4% for the non-metformin group, p = 0.01)^19^.

Previous studies have attempted to identify the effect of metformin on OS and DFS in CRC patients. However, the results were considerably different. In a register-based observational study of 1 116 diabetic CRC patients in Denmark, they found no association between metformin use and DFS or recurrence-free survival in patients who underwent surgery for CRC after adjusting for confounders^12^. However, in this study, only patients medically treated for diabetes were classified as diabetic. Indeed, there are about 2 million diabetic patients in France, and more than 200,000 to 300,000 are treated by diet alone^20^.

One of the antineoplastic action of metformin is based on the epithelial-mesenchymal transition (EMT) activity^21^. EMT occurs when epithelial cells lose their epithelial characteristics, including polarity and adhesion, and develop migratory properties by transformation into mesenchymal cells. In cancer, invasion, recurrence and metastasis are associated with EMT^22^. E-cadherin is an inter-cellular adhesion marker. Its loss demonstrates activation of EMT, thus witnessing tumor aggressiveness in CRC^3^. An ex-vivo study showed an increase of E-cadherin expression in CRC tissues in diabetic patients with CRC using metformin in comparison to metformin non-users, and better OS and DFS for metformin users^23^.

Our study was able to highlight further interesting elements. In subgroup analysis, we found that the use of metformin in combination with other antidiabetic drugs was not associated with better DFS compared with metformin users alone. This may be due to a possible antagonistic effect between metformin and the other antidiabetic drugs^24^.

We also performed a subgroup analysis according to cancer location. We found that the protective effect of metformin was detected among patients with colon and rectal cancer subgroups. Results obtained by Lee et al*.* showed that survival benefits of metformin use were present for rectal cancer, but not for colon cancer^25^. CRC has different clinical characteristics and tumorigenic pathways depending on tumor location, including different molecular pathways, microsatellite stability, and prognosis^26^. Therefore, more studies targeting cancer localization and CRC survival of metformin users should be conducted.

While DFS was significantly higher in metformin users regardless of CRC stage, subgroup analysis revealed that metformin use was protective in stages II and III CRC.A previous study demonstrated an attenuated association between metformin use and CRC–specific survival after including patients with stage IV CRC in their study^27^. These results suggest that metformin has a protective effect in patients with early-stage CRC. However, this analysis was limited by a small simple size.

Among metformin users, having a BMI ≥ 25 was not associated with DFS compared with a normal BMI (18–24). This suggests that metformin acts through a glucose-independent pathway, as it was shown in an in vitro study focusing on metformin and stomach cancer^28^. This finding is of high importance and may query about the potential therapeutic effect of metformin not only on diabetic patients but also in non-diabetic ones. However, further in vivo evidence on CRC should be conducted.

To explore the effect of microsatellite instability on the relation between CRC survival and metformin, we examined the association between DFS and metformin use in MSI and MSS subgroups. The protective effect of metformin was detected among subgroup of MSS patients and not MSI. A study conducted on patients with resected stage III colon cancer found no difference in DFS, OS and time to recurrence between metformin users and non-users for both defective DNA mismatch repair (dMMR) and proficient DNA mismatch repair (pMMR) tumors^29^. Because of the low frequency of MSI in CRC, few studies on the use of metformin and MSI have been reported in CRC. Hence, further studies are needed to detect any significant effects’ difference of this medication regarding MMR status.

Despite a large number of CRC patients with type 2 DM, the number of patients was small in some subgroups, which limited the ability to detect small differences between metformin users and non-users within some subgroups. Consequently, subgroup results should be interpreted with caution.

This study has several strengths, including the availability of a large French cohort operated for CRC over a long period of 14 years. In addition, patients were operated for CRC including all stages (from I to IV). The fact that it was a monocentric study implies same environment and same oncological management avoiding thus some biases^13^. The demographic, clinical, drug use and laboratory findings were available allowing to control for multiple potential confounders in the multivariate analysis and the effect of metformin on OS and DFS has remained significant.

Because metformin is the first-line pharmacological treatment for type 2 DM, and is therefore used in patients with early diabetes, we may see better survival among metformin users who are considered healthier than non-users of metformin (healthy user bias 38). Furthermore, complications associated with diabetes may also lead to an increased risk of mortality. In our study, this confounding bias was reduced by adjusting for diabetes complications. In addition, because metformin users were younger than non-users, we stratified the data by age of 74 years, to confirm that metformin use is the main cause of the better survival and rule out the effect of age. In stratified and multivariate analysis, metformin users still have a better DFS in both age categories; younger and older than 74 years. This means that the better survival observed in metformin users is not due to the younger age of metformin-users given its protective effect detected in both younger and older patients separately. However, the study presents some limits. Information bias may have occurred due to the retrospective nature of the study. The analysis was limited by the lack of information on the effect of time-dependent exposure and the duration of diabetes, therefore, their effects could not be examined which increased the effects of time-related biases, such as the immortal time and time lag bias, which were also present in some previous studies^30,31^.

Although we focused on OS in our study, we recognize that cancer-specific survival would have been more relevant. However, the cause of death was unavailable due to limitations in death certificate information. In addition, the source of mortality was limited to the death certificate available in the hospital's cross-crossway system, this could underestimate the events that occurred.

A possible selection bias may be present, because only patients admitted to Limoges University hospital were included which may have more advanced stages and more comorbidities compared to the patients in other centers but to reduce these effects, we adjusted to the CRC stage and Charlson comorbidity index in our multivariate analysis.

The improved survival observed in metformin-users needs to be confirmed in larger prospective cohorts of people with diabetes over an extended follow-up period, considering time-related drug-exposure and applying the propensity score method. Randomized controlled trials are also needed to further evaluate the survival benefit of metformin use.

## Conclusion and perspectives

Our data suggest that the use of metformin may improve OS and DFS in CRC patients with type 2 DM. Interesting findings have been underlined especially the beneficial role of metformin for early CRC stages and MSS status.

## Data Availability

The data presented in this study are available on request from the corresponding author (Z.T.).
